# Medical Opinion and Movement

**Published:** 1909-04-17

**Authors:** 


### Medical Opinion and Movement.

It will be remembered that, as a result of his researches on coagulation, Wright suggested the administration of sodium citrate with the milk for infants, with a view to inhibit the coagulation and cause the formation of small clots after the manner of human milk, instead of the usual bulky clots of cow's milk. Dr. Gaucher, of Montpelier, has been conducting a large amount of research on the digestion of milk, and he finds that sodium citrate, even in doses of 8 grammes per litre, does not impede coagulation in the stomach. According to this author, if rennet be used to coagulate the milk *in vitro*, citrate of sodium will inhibit coagulation for several hours; but if gastric juice itself be used, even in much less quantity than is ordinarily secreted by the stomach during the digestion of the milk, sodium citrate has no inhibiting effect at all. He gets the same results by substituting sodium fluoride for the sodium citrate—inhibition in the case of rennet, but no effect with gastric juice. He suggests that the gastric juice brings a further quantity of calcium to the milk which is sufficient to cause its coagulation. It would appear, therefore, according to this author, that such anticoagulants as sodium citrate have no therapeutic value in the way that has been suggested.

---

In a communication to the Society of Physicians of Vienna, Dr. Exner draws attention to the fact that the position of the heart between the lungs enables it to get rid of the over-production of heat which necessarily accompanies its continual work. The lungs act as coolers to the heart, which would otherwise tend to be overheated. The work of the heart amounts to 10,000 kilogrammetres per day, of which about two-thirds are transformed into heat, giving approximately 70 calories, whereas, according to the weight of the heart, it should not produce more than 13 calories. Yoshimura has carried out some observations on the temperature of the heart and lungs in animals, and finds that the temperature of the ventricular wall exceeds that of the lungs by 0.5° C. The temperature of the lungs diminishes in proportion to the distance from the heart. He finds, too, that the ventricular wall has a higher temperature than the blood it contains, amounting to 2° C. Moreover, the blood in the right ventricle is hotter than that in the left. In the latter case it has been presumably cooled by its passage through the lungs. By surrounding the heart with cotton-wool, so as to prevent the conduction of heat to the lungs, the temperature rises, the heart-beats become more rapid, and then cease. If the cotton-wool is removed, the heart contracts again. Although it may be correct to assume that the position of the heart beween the lungs facilitates the dispersion of excessive heat produced by the heart, these experiments would also appear to indicate that a large proportion of this heat is carried off by the blood circulating within it. While it is shown that there is a difference of 0.5° C. between the ventricular wall and the lungs, the much greater difference of 2° C. between the heart and the contained blood suggests that the dispersion of excessive heat from the heart takes place more especially in this direction.

---

In *La Clinique de Paris* Dr. Sersiron makes a contribution on the sun-bath, and speaks very enthusiastically of its therapeutic effects in all forms of tuberculosis and in cases of lymphatism and anæmia. In Germany this mode of treatment is in great repute. It is stated that in the environs of Dresden every Sunday 3,000 persons indulge in a sun-bath, in the costume of Adam, at the cost of 50pf. a head. In France it is not popularised in this way, but the treatment is carried out under medical supervision at several "solariums" on the Riviera, and the results obtained appear to be highly satisfactory. The treatment must be gradual and progressive. At first only the part affected with disease is exposed to the sun's rays for a few minutes. Solar dermatitis must be avoided, but as soon as the skin has reacted by the development of pigmentation, exposures of several hours can be made. It is observed that the greater the pigmentation of the skin, the more favourable is the reaction of the patient to the treatment. The author quotes several statistics showing the favourable results that have been obtained by the treatment. The duration of the treatment is considerable. Tuberculous patients of the first degree require a year, and more severe cases 18 months to two years. The action of the rays is probably of a mixed nature. It is suggested that it is in part electric, as every form of heat is accompanied by the production of electricity. The red rays are excitants of the nervous system and are tonic; they are vasodilators, and produce a passive congestion analogous to that of the method of Bier. The yellow and green rays are supposed to influence the red corpuscles similarly to the action of the red rays upon the chlorophyll of plants. The blue and violet chemical rays are destructive of the ferments, diastases, the microbes and their toxines, and the ultra-violet rays probably have a similar action. Such is briefly the hypothetical explanation of the therapeutics of the sun-bath. Of its efficacy the author is evidently thoroughly convinced. Unfortunately, climatic conditions are not favourable in this country for the practical adoption of the treatment.

---

At a recent meeting of the Academy of Medicine of Paris, Dr. Chantmesse made an interesting communication on the coagulation of the blood in certain morbid conditions, and the occurrence of fatal embolism, especially after operations for the removal of uterine fibromata. It is a well-established fact that the coagulability of the blood varies under different conditions, and it has been shown that this is often increased, for instance, in typhoid fever. Wright explains this increase as caused by an excessive amount of calcium salts in the system owing to the milk diet, and suggests the administration of citric acid to counteract the effect. He has definitely shown that the coagulability of the blood can be diminished by the administration of citric acid, and increased by calcium salts. Dr. Chantmesse suggests however, that there is a further physiological factor in the determination of the degree of coagulability of the blood—namely, the reaction of the organism to hæmorrhage. Continual loss of blood, as in the case of uterine fibroids, or in some cases of typhoid fever induces spontaneously an increase of coagulability as a protective measure. When added to this increased coagulability there is a stagnation of the blood in the veins, thrombosis and embolism are likely to intervene. It is in this way that the author seeks to explain the frequent occurrence of fatal embolism after removal of uterine fibroids. He advises, therefore, that an estimation of the coagulability of the blood should be made both before and after operation in these cases, and that, if necessary, citric acid should be given in doses sufficient to reduce it to a normal degree. He thinks in this way such untoward accidents might be successfully avoided.

---

EXPERIMENTS with Digitalis on the Frog's Heart and its homologues seem to have convinced Pohl that the association of such drugs with others in a mixture does more harm than good. He publishes his conclusions in the *Therap. Monatshefte*. He finds that all acid drugs destroy the action of digitalis; while quinine retards the effects of the contained glucoside. If potassium salts are prescribed along with strophanthin to increase diuresis, they exert an inhibitory action on the latter drug. On the other hand the action of strophanthin is intensified by the addition of an ammoniacal solution of anise, as it is also by chloroform water. Tincture of opium moderates the effects of the drug, and caffeine has a marked inhibitory action. The author is therefore of opinion that if rapid results are desired, digitalis and its congeners must be prescribed by themselves.

---

AN interesting article from the pen of Miramont de Laroquette appears in a recent number of *La Revue Médicale de l'Est*, dealing with the therapeutic and hygienic aspects of the Incandescent Electric Lamp. According to the author this lamp emits rays whose action is in every way similar to those of the sun. They increase the rapidity of growth of plants, while they arrest the development of and eventually kill such lowly forms of vegetable life as bacteria. In men and other animals they provoke a marked hyperæmia with erythema, sweating, and increased cellular activity, and in addition have an analgesic action. A temperature of 150° can be imparted to the surrounding air by the lamps. These light baths have a sedative action on the nervous system, and lower the blood pressure. They are especially useful in the treatment of chronic inflammation, the sequelæ of acute infections, as also in chronic rheumatism and gout. The author recommends their use in acute conditions which are accompanied by subnormal temperature; also where rigors are present and in conditions of profound shock such as are observed after intestinal perforations, and in the various choreiform affections. Post-operative shock and paralysis of the intestine after laparotomy are also benefited by these baths. The active hyperæmia induced by the rays facilitates the resorption of inflammatory exudates, softens scars, and even promotes the dissipation of fibrous new growths, while the processes of repair are awakened in indolent wounds and ulcers. The analgesic action of the rays is particularly useful in the treatment of neuralgia and painful chronic diseases. Among other affections treated by this method may be mentioned obesity, arterio-sclerosis, and neurasthenia. Many observers claim to have cured cutaneous disorders such as psoriasis by the rays, and Minirow has published a case of lupus cured by their means.

---

A NEW method for the Diagnosis of Gastric Ulcer is described by Bonninger in the *Berliner Klinische Wochenschrifte*. It is well known that one of the most important symptoms of this malady consists in the onset of pain about half an hour after taking food. This pain is attributed to the gradual acidification of the stomach contents, and it increases in severity *pari passu* with the intensity of the acidity. Relying on these facts the author has evolved a method of diagnosis whereby the stomach is thoroughly washed out with plain water in the early morning when the patient is fasting; the washings are then removed and about 100 c.c. of a 5 per cent. solution of hydrochloric acid is introduced into the organ. If a gastric ulcer is present the patient is promptly seized with violent pain in the stomach. This is immediately relieved by the administration of a little milk. If, however, no pain is felt the patient is made to change his position so that the acid may come into contact with every part of the mucous surface of the stomach. Should no pain declare itself after this manœuvre it is concluded that no ulcer exists. Obviously this method only serves to render the pain, common to this affection, more characteristic, and it cannot therefore be applied in cases in which no pain is present, even when the stomach is in the full activity of digestion. It must, however, be noted that the acid solution causes no pain in healthy persons nor in those suffering from gastric disease other than ulcer. The author further claims that by his method the progress of cicatrisation of the ulcers can be followed. We can, however, imagine various objections to a means of observation which involves repeated bathing of the injured and highly sensitive gastric wall with an irritant acid.

---

THE curious condition known as Multiple Hereditary Telangiectasies (Osler), and also as Telangiectasis Circumscripta Universalis has been prominent in medical literature during the last year or two. In fact, during the last three years eight such families have been reported, as against but six previously recognised in all. The most recent of these are two families described in the *Johns Hopkins Hospital Bulletin*, by Dr. Hanes. In one of these, out of fourteen individuals who comprise the last four generations, nine are sufferers from hereditary telangiectases; in the other, four sisters are affected out of a family of nine, whose father transmitted to them the tendency. Appended to the details of these families are abstracts of all the other cases so far published, and a summary of our knowledge of the lesion, which seems to be less rare than has until recently been supposed. This summary carries us rather further than Professor Osler's similar survey of the condition in the *Quarterly Journal of Medicine* of October 1907. The disease is defined as an hereditary affection manifesting itself in localised dilatations of capillaries and venules or telangiectases, particularly common on the face, nasal and buccal mucosæ, and finger-tips. Its most striking characteristic, next to the very markedly hereditary nature of the lesion, is its tendency to set up profuse hæmorrhages, either spontaneously or as the result of trauma. These hæmorrhages are often of alarming severity, and have in some of the reported instances caused death.

At the same time it is clearly to be understood that the condition is totally distinct from hæmophilia. Thus arthropathies, hæmaturia, and melæna are never found. It does not, as hæmophilia does, affect males only; nor is the tendency transmitted through the unaffected females. It appears equally in males and females (some think more frequently in the latter), and can be transmitted by either parent. It is said that there is no instance in the literature of an affected patient having children all of whom have been free from the disease. Repeated traumata are believed to play a part in the production of telangiectases, and in the rupture of those already formed by such trivial acts as blowing the nose, eating, and so on. Another ætiological factor to which Hanes is inclined to attach importance is the abuse of alcohol; but it must be added that Osler does not mention such a factor. Epistaxis is the commonest form of hæmorrhage. The condition has been found as early in life as eight years old, but usually begins to show itself in the third decade. As regards treatment, Professor Osler thinks calcium lactate did one patient a great deal of good, though another one under his care got no benefit from the chloride of that metal. Apart from this, cauterisation of the individual lesions seems to be the only way to relieve the condition. This Hanes advocates by the use of chromic acid, applied to every telangiectasis in turn, at several sittings if necessary. This author proposes yet a third name for the disease (which he regards as a definite clinical entity), different from the two it has already received. But his suggestion of hereditary hæmorrhagic telangiectasia differs so little from Professor Osler's name that the change seems unnecessary.

A case of Tobacco Amblyopia from cigar-smoking by a woman is reported in the *California State Journal of Medicine*. The patient was fifty-two years of age, and a woman of education and refinement. She consulted Dr. W. S. Franklin, of San Francisco, for failing sight, which she had noticed coming on gradually for some time, but had attributed to her advancing years. On examination it was found that vision was reduced to finger-counting at five feet in both eyes, that an absolute central scotoma for red and green existed, and a relative scotoma for form. When questioned as to alcohol and tobacco she denied the former absolutely, but admitted at once that she had been smoking six to eight cigars a day for two years past. The author thinks it is quite certain that the answer about alcohol was a truthful one. On ophthalmoscopic examination the discs were found to be distinctly pale, but no other ocular abnormality existed. In six months vision was restored to $\frac{6}{6}$ with a correcting cylinder of one dioptre, and to $\frac{6}{9}$ without it. This happy result Dr. Franklin regards as additional proof that the amblyopia was purely due to tobacco, as the prognosis in toxic amaurosis due to mixed tobacco and alcohol poisoning is much less favourable. He remarks that cases of pure tobacco amblyopia, such as this, are rare in women, who, as is well known, suffer much less frequently from these lesions than do men. Yet another curious feature about the case was the indulgence in cigars alone: cigarettes and pipes had never been used. It is, as the author points out, extraordinary how little attention is bestowed even by intelligent people on progressive failure of their sight almost to blindness, so long as it is painless and gradual in onset.

Dr. Seymour Taylor, in a lecture on Epilepsy printed in the *West London Medical Journal*, emphasises the instability of cortical centres other than the motor in this disease. Not only may the chief lesion be in the psychical or sense areas of the cortex, it may even be in the cerebellum, the optic thalamus, the medulla, or the spinal cord. Moreover, wherever the principal lesion is, the effects of its "hyperphysiological discharge" spread rapidly to other centres which, in the case of true epilepsy, are similarly but less severely affected. On this hypothesis the "aura," be it gustatory, visional, auditory, psychical, or what not, is the beginning of the fit; and petit mal is epilepsy of some psychical centre or centres which passes off without extending to the motor cortex. On this hypothesis also Jacksonian epilepsy is explained, for this is due to local irritation of the motor cortex by growth, blood clot, depressed bone, or other gross lesion, which can so far upset the stability of the cells affected as to provoke discharge there, but is powerless to cause spread to the neighbouring healthy cells not irritated. Dr. Taylor describes cases which he has diagnosed as epilepsy of the psychical centres alone, in some of which the ordinary manifestations have subsequently appeared. One patient was subject without warning to long trains of peculiar thoughts which were unconnected with each other and with her occupation at the moment; she was during these attacks unable to proceed with reading or anything else she might be doing, and was in fact only semi-conscious. Other patients have roaring in the ears, distressing taste sensations, or other "auræ," without the slightest mental confusion; these patients are benefited by bromides. Nocturnal enuresis has long been recognised as occasionally due to unsuspected epilepsy, and Dr. Taylor thinks that some instances of tumultuous and irregular cardiac action without discoverable cause have also a similar foundation.

